# Mechanistic Insight into the Mode of Action of Acid β-Glucosidase Enhancer Ambroxol

**DOI:** 10.3390/ijms23073536

**Published:** 2022-03-24

**Authors:** Supansa Pantoom, Larissa Hules, Christopher Schöll, Andranik Petrosyan, Maria Monticelli, Jola Pospech, Maria Vittoria Cubellis, Andreas Hermann, Jan Lukas

**Affiliations:** 1 Translational Neurodegeneration Section “Albrecht-Kossel”, Department of Neurology, University Medical Center Rostock, 18147 Rostock, Germany; supansapantoom@yahoo.com (S.P.); larissa.hules@uni-rostock.de (L.H.); andreas.hermann@med.uni-rostock.de (A.H.); 2 Leibniz Institute for Catalysis, University of Rostock, 18059 Rostock, Germany; christopher.schoell@ur.de (C.S.); andpharm@gmail.com (A.P.); jola.pospech@catalysis.de (J.P.); 3 Department of Biology, University Federico II, 80126 Napoli, Italy; maria.monticelli@yahoo.com (M.M.); cubellis@unina.it (M.V.C.); 4 Istituto di Chimica Biomolecolare—CNR, 80078 Pozzuoli, Italy; 5 Center for Transdisciplinary Neurosciences Rostock (CTNR), University Medical Center Rostock, University of Rostock, 18147 Rostock, Germany; 6 German Center for Neurodegenerative Diseases (DZNE) Rostock/Greifswald, 18147 Rostock, Germany

**Keywords:** Gaucher disease, small molecule therapy, pharmacological chaperone, drug repositioning, lysosomal storage disease, rare disease, thermal shift assay

## Abstract

Ambroxol (ABX) is a mucolytic agent used for the treatment of respiratory diseases. Bioactivity has been demonstrated as an enhancement effect on lysosomal acid β-glucosidase (β-Glu) activity in Gaucher disease (GD). The positive effects observed have been attributed to a mechanism of action similar to pharmacological chaperones (PCs), but an exact mechanistic description is still pending. The current study uses cell culture and in vitro assays to study the effects of ABX on β-Glu activity, processing, and stability upon ligand binding. Structural analogues bromohexine, 4-hydroxybromohexine, and norbromohexine were screened for chaperone efficacy, and in silico docking was performed. The sugar mimetic isofagomine (IFG) strongly inhibits β-Glu, while ABX exerts its inhibitory effect in the micromolar range. In GD patient fibroblasts, IFG and ABX increase mutant β-Glu activity to identical levels. However, the characteristics of the banding patterns of Endoglycosidase-H (Endo-H)-digested enzyme and a substantially lower half-life of ABX-treated β-Glu suggest different intracellular processing. In line with this observation, IFG efficiently stabilizes recombinant β-Glu against thermal denaturation in vitro, whereas ABX exerts no significant effect. Additional β-Glu enzyme activity testing using Bromohexine (BHX) and two related structures unexpectedly revealed that ABX alone can refunctionalize β-Glu in cellula. Taken together, our data indicate that ABX has little in vitro ability to act as PC, so the mode of action requires further clarification.

## 1. Introduction

The pharmacological chaperones (PCs) represent a novel form of therapy for diseases involving protein misfolding, such as lysosomal storage disorders (LSDs). PCs specifically bind to folded or partially folded proteins to provide enhanced stabilization and enable trafficking of mutant but intrinsically functional protein to the target organelle [1,2]. Most PCs are reversible competitive inhibitors of their target protein, even though the inhibitory activity is not desired [3]. The development of efficient PCs is an unmet need for all LSDs as the efficacy of existing therapies (e.g., enzyme replacement therapy and substrate reduction therapy) is limited by their inability to cross the blood-brain barrier or by undesirable side effects.

Gaucher disease (GD; MIM# 230800, 230900, 231000) is a rare autosomal LSD caused by mutations in the *GBA1* gene (MIM# 606463) encoding for the lysosomal enzyme acid β-glucosidase (β-Glu, EC 3.2.1.45). GD has an estimated incidence of 1/40,000 in the general population and an incidence of 1/1000 in the Ashkenazi Jewish population [4]. The resulting decrease in enzymatic activity leads to a progressive accumulation of glucosylceramide and glucosylsphingosine in lysosomes, causing organ enlargement, anemia, and bone disease (Type I) and additional neurological impairment (Types II and III) [5]. A range of different *GBA1* gene variants, including the prevalent p.Asn370Ser allele associated with Type I GD, have been tested for their response to various PCs [6,7,8], so GD can be considered a reasonable target for PC development. One such PC, the iminosugar isofagomine (IFG), selectively inhibits β-Glu in cell lysates in a competitive manner, with IC_50_ values of 30 (5) nM (wildtype β-Glu) and 128 (18) nM (p.Asn370Ser variant) at pH 5.2 (7.2) using 3 mM of 4-methylumbelliferyl-β-D-glucopyranoside (4-MUG) [9]. A similar result was obtained for wildtype β-Glu in a later study using recombinant enzyme [1]. A six-month Phase 2 clinical trial was carried out in 19 adults with Type I GD, but the drug was not advanced to Phase 3 development due to a lack of meaningful reduction in disease symptoms in the majority of the patients [10]. It can be assumed that potent competitive inhibitors harbor the risk of interfering with the natural reaction conditions in the cells in an adverse manner. Thus, it may seem that the development of either allosteric, non-inhibitory, or less-potent isosteric PCs is imperative to comply with safety demands in the drug approval process.

Around the same time as IFG failed in clinical trials, the substituted benzylamin ambroxol (trans-4-(2-amino-3,5-dibromobenzylamino)-cyclohexanol (C_13_H_18_Br_2_N_2_O, ABX) was identified by thermal denaturation assay [11]. In this study, recombinant β-Glu was subjected to heat treatment at 51 °C for 12 min, and it was found that ABX-treated β-Glu exhibited higher enzyme activity compared to untreated enzyme. The evident advantage of this assay was that ABX was detected by means of its β-Glu enzyme stabilizing effect rather than β-Glu inhibition. Maegawa and colleagues further investigated the inhibition mode and binding site and characterized ABX as a mixed-type inhibitor that exerts its inhibitory effect in the micromolar range [11]. It is of note that ABX is a derivative of BHX; however, BHX did not prove to be a PC but exhibited inhibitory function towards β-Glu [1,11,12]. Numerous studies followed in which patient fibroblasts with different mutations, e.g., p.Arg120Trp, p.Phe213Ile, and in some cases p.Leu444Pro, responded significantly positively to ABX exposure [13,14,15]. The positive effects could be reproduced in other cell and animal models, such as mouse and fruit fly [16,17,18,19]. ABX treatment achieved a positive outcome on blood parameters and organ volume in a pilot study of six previously untreated Type I GD patients and was well-tolerated [20]. Later studies concluded with a similarly promising result in Type III GD patients using ABX as either mono- or combination treatment with enzyme replacement therapy [21,22]. 

Successive studies suggested that ABX does not act solely as a PC but also at the cell biological level as a transcriptional regulator, for example by increasing *GBA1* and transcription factor *TFEB* mRNA [23,24] and the transcription of the ER stress-related transcription factor CHOP [19,25], which are thought to play a role in the expression process of β-Glu [26,27]. However, no conclusive link has been established between chaperoning and the influence on cellular physiology. Similarly, in studies on Fabry disease, a beneficial effect of ABX on mutant α-Galactosidase A (α-Gal) was observed, but the mechanism of action remained obscure [28,29]. Nevertheless, ABX was not efficient in single application, whereas combinatory treatment indicated a PC-supportive effect. Furthermore, the study by Seemann et al. [29] demonstrated that the efficient α-Gal enhancer compounds each showed both an inhibitory effect on the proteasome and a positive gene regulatory effect on the α-Gal target gene itself. Neither effect could be shown for ABX. Since both the α-Gal and β-Glu data do not currently allow the enzyme-enhancing effect of ABX to be mechanistically termed “chaperoning”, in the present work we have studied the effects of ABX and IFG on β-Glu in various biochemical and cell biological experiments. There are remarkable differences in the processing and stability of the enzyme under IFG and ABX influence in cells, and the investigation in a quantifiable in vitro β-Glu thermostability assay indicated a much weaker ABX-mediated stabilization effect. 

## 2. Results and Discussion

### 2.1. β-Glu Enzyme Is Weakly Inhibited by Ambroxol

**Figure 1:**
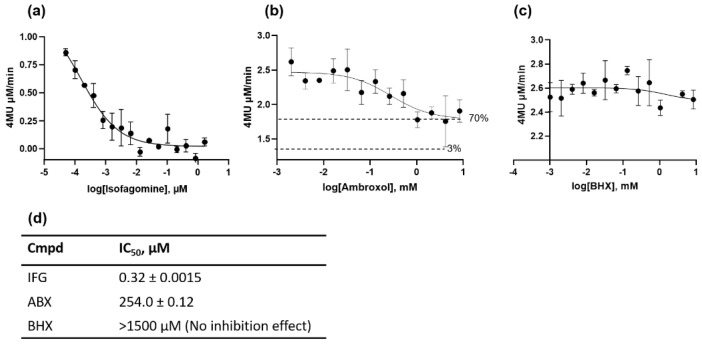
Inhibition of recombinant acid β-glucosidase (β-Glu). Endpoint enzyme activity measurement was recorded in the presence of different concentrations of isofagomine (IFG) (**a**), ambroxol (ABX) (**b**), and bromohexine (BHX) (**c**) after a 20 min incubation phase using 1.5 mM 4-methylumbelliferyl-β-D-glucopyranoside (4-MUG) synthetic substrate at pH 4.7. (**d**) IC_50_ was calculated from the dose–response curves using the function “log(inhibitor) vs. response” (GraphPad Prism 5 for Windows, GraphPad Software, La Jolla, CA, USA).

Most of the identified PCs for lysosomal hydrolases are isosteric inhibitors. They are biosimilar sugar compounds of the natural substrate. The benzylamin compounds ABX and BHX are an exception among the inhibitor structures for β-Glu. All these structures have in common the aim to increase the stability of both normal and mutant enzyme forms. In this way, the activity of functionally compromised enzyme variants can be recovered. Unlike IFG, which was confirmed as a competitive inhibitor, the mode of action for ABX and BHX was characterized as mixed inhibition [1]. In this study, we reproduced the inhibitory activity of the substances. The half-maximal inhibitory concentration (IC_50_) of the active site-inhibitor IFG was 0.32 ± 0.0015 nM at pH 4.7 when 0.026 µM recombinant β-Glu was used and at a substrate concentration of 1.5 mM 4-MUG (Figure 1a,d). The considerable dependence on pH, enzyme-substrate ratio, and other parameters such as buffer substance were not considered in our experiment. The same applies to the other experiment series. For ABX, an inhibition effect was measured that was almost three orders of magnitude lower under the applied conditions, with an IC_50_ of 254.0 ± 0.12 µM (Figure 1b,d). The half-maximal inhibitory concentration represents the concentration of an inhibitor that is required to reduce enzyme activity by 50%. For ABX, we defined it as the concentration at which the half-maximal inhibitory effect was achieved, since the inhibition by ABX was incomplete (as known from previous studies) [12,30]. In the current experiment, the residual enzyme activity was about 70%. Remarkably, BHX had no significant inhibitory effect in the tested concentration range (Figure 1c,d). While it has been suggested that the strength of the inhibitory effect has a positive predictive value for the efficiency of the chaperone effect [31], robust data for this hypothesis are not available. At this point, the statement can once again be supported that IFG is a strong inhibitor while ABX is a weak inhibitor. It should be noted that IFG shows a proven altered kinetics with mutated enzyme [9], which can also be assumed for ABX as different binding affinities have been calculated using molecular dynamics simulation for different drugs to the abnormal enzyme variants [32].

### 2.2. Enzyme Activity Is Enhanced in Cell Culture

**Figure 2:**
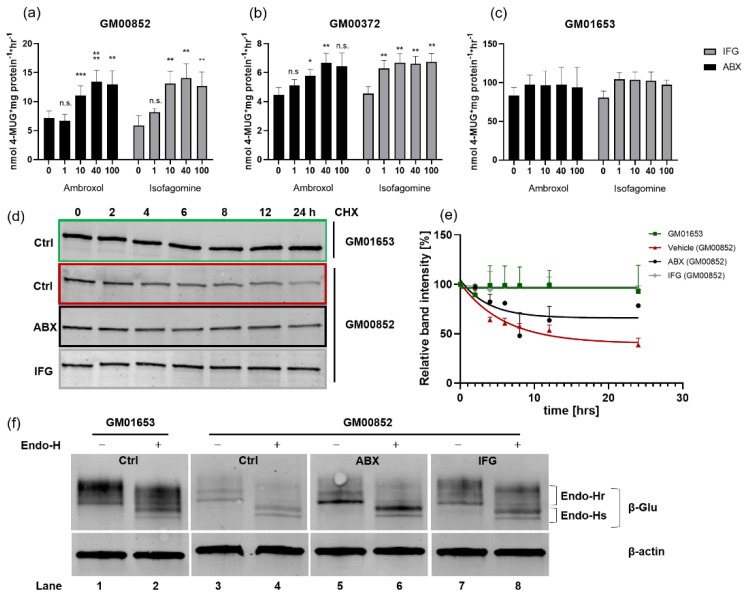
Acid β-glucosidase (β-Glu) enzyme activity, stability, and glycosylation in compound-treated patient fibroblasts. (**a**–**c**) β-Glu activity in ambroxol/isofagomine (ABX/IFG)-treated cells. Three cell lines of unrelated patients harboring either compound heterozygous p.Asn370Ser/84GG (GM00852, GM00372) mutations or wildtype *GBA1* (GM01653) were cultured for 5 days in absence and presence of ABX (black columns) or IFG (grey columns). The cells were harvested following a 6 h washout period, lysed, and subjected to enzyme measurement using 3 mM 4-methylumbelliferyl-β-D-glucopyranoside (4-MUG). Data are provided as mean ± SEM from 6–12 (**a**,**b**) and 3 (**c**) independent experiments. Differences between the groups were analyzed using one-way ANOVA. Post-hoc Dunnett test was used to analyze each column with the respective value of the untreated cells (significance level is represented by *, **, *** as *p*-values of 0.05, 0.01 and 0.001); n.s., not significant. (**d**) Cycloheximide (CHX) chase experiment. Patient fibroblasts of line GM00852 and control cells (GM01653) were cultured for 5 days in the presence or absence of treatment. Then, in addition to treatment, CHX was added for at 0, 2, 4, 6, 8, 12, and 24 h to study intracellular stability of β-Glu. Cell lysates containing 20–30 μg were digested with PNGase F to obtain a singular protein band in immunoblot using anti-β-Glu (2E2) antibody. (**e**) Decay curves of β-Glu in fibroblast cells. Each β-Glu band was normalized to its corresponding β-Actin band (not shown). All normalized values obtained were referenced to the 0 h time point of each treatment series before CHX chase start (100%). The values are provided as mean ± SD from 2–3 independent experiments. For each treatment regimen, a nonlinear fit analysis of the resulting values was performed. (**f**) Glycosylation analysis of cellular β-Glu. Cell lysates of ABX, IFG or control-treated fibroblasts were subjected to Endo-H digestion and Western blot. Qualitative band analysis was repeated in two independent experiments: Endo-Hr, Endoglycosidase H-resistant; Endo-Hs, Endoglycosidase H-sensitive.

The p.Asn370Ser mutation is the most common GD associated mutation. It is present in over 70% of Ashkenazi Jewish Type I GD patients (who are either homoallelic or heteroallelic with another GD mutation) [33]. We analyzed the PC response in two p.Asn370Ser/84GG compound heterozygous cell lines: GM00852 and GM00372. The two cell lines exhibited β-Glu enzyme activity of 8.3 (±1.4) nmol 4-MUG × mg protein^−1^ × h^−1^ and 5.0 (±0.5) nmol 4-MUG × mg protein^−1^ × h^−1^, respectively. The wildtype cell line used for comparison, GM01653, exhibited β-Glu activity of 83.6 (±10.3) nmol 4-MUG × mg protein^−1^ × h^−1^ (Figure 2a–c). The reported activity of the p.Asn370Ser mutant ranges from 14% to 30% [6,33]. The somewhat lower percentage activity of the two GD lines under investigation here might be explained by the fact that the second allele is a NULL allele carrying the nonsense mutation 84GG [34]. ABX and IFG treatment of the cells for 5 days plus a short washout step of 6 h showed significant responsiveness of β-Glu in both GD lines (Figure 2a,b) and maintenance of activity in the WT cells (Figure 2c).

GM00852 cells showed increases of up to 14.1 (±2.5) nmol 4-MUG × mg protein^−1^ × h^−1^ and 13.5 (±1.9) nmol 4-MUG × mg protein^−1^ × h^−1^ after treatment with IFG and ABX, respectively, at the most effective concentrations of 40 µM each (Figure 2a). GM00372 had elevated β-Glu activity up to 6.7 nmol 4-MUG × mg protein^−1^ × h^−1^ for IFG and ABX at 100 µM and 40 µM, respectively. In a previous study on a series of deoxynojirimycin derivatives as PC candidates, the two GD lines showed maximum responsiveness of 1.7-fold (GM00372) and up to 2.4-fold (GM00852) after 4 days of treatment [35]. In other studies with cells carrying the p.Asn370Ser mutation, similar relative increases were also observed after application of different molecular drugs [7,35,36,37,38,39]. The values are generally in the same range as the fold changes from the analyses carried out here (Table 1). IFG showed a weak, insignificant β-Glu chaperoning activity of 1.3-fold (1–100 µM) on the wildtype enzyme in GM01653 cells. ABX also barely affected the activity (1.1-fold increase at 5–40 µM). However, it is an important indication of the safety that normal activity is not hampered by either of the PC treatments. Specifically, PCs for β-Glu might be a potential drug in combating Parkinson’s disease [13,40] due to the association between mutations in the *GBA1* gene and an increased risk of early Parkinsonism [41,42]. These patients carry the WT allele in addition to a mutant *GBA1* allele. It has previously been demonstrated that cells carrying the p.Leu444Pro mutation are amenable to ABX [11,14]. We did not intend to reproduce these results in this study because we concluded that the positive effects of ABX and IFG on mutant β-Glu activity in cell culture has been sufficiently verified. Another indication of the safety of the treatment is the result of the MTT viability assay (Supplementary Materials Figure S1a). Only high ABX concentrations (>40 µM) showed slightly toxic effects in all three cell lines in vitro. Since it is known that even long-term treatment with a high dose of ABX of up to 21 mg/kg/day is well-tolerated by the organism [43], it cannot be excluded that the ABX toxicity here is a cell-culture-specific outcome.

### 2.3. Differential β-Glu Stability and Processing Level Following ABX and IFG Treatment

Treatment with active-site-occupying compounds leads to a prolongation of the half-life of the mutant enzyme [44]. To determine whether stabilization of β-Glu in GM00852 is equally efficient, we cultured the cells for 5 days with or without addition of ABX or IFG. Thereafter, the cells were treated with the protein translation inhibitor cycloheximide (CHX) for an additional 2, 4, 6, 8, 12, and 24 h to prevent de novo protein synthesis. PC treatment was continued in each case. We found that ABX was able to slow intracellular β-Glu degradation compared to the native state (Figure 2d,e). After 24 h, 61.5% of native β-Glu (Figure 2e, red curve) was degraded. ABX-treated intracellular β-Glu showed degradation by 21.5%, less than half of the untreated enzyme (black curve). IFG was so efficient that after 24 h hardly any protein degradation was detectable (gray curve). The wildtype enzyme in GM01653 cells (Figure 2e, inlayed picture, green curve) also showed stable enzyme levels and insignificant β-Glu degradation. 

Lysosomal enzymes are synthesized in the ER. N-glycan conjugation allows further transport to the Golgi apparatus, where sugar-chain modification leads to the formation of high-order complex carbohydrates. Endoglycosidase H (Endo-H) cleaves high-mannose but not complex oligosaccharides. The Endo-H reaction therefore identifies post-Golgi processed protein by causing Endo-H sensitive (Endo-Hs) protein to undergo a size shift, whereas Endo-H resistant (Endo-Hr) protein does not change size. Thus, indirect statements about the cellular localization of proteins can be made. After 5 days of culturing GD fibroblasts with the appropriate treatment and harvesting without a washout period, cell lysates were obtained. The results of this experiment are shown in Figure 2f. The first finding is that mutant p.Asn370Ser β-Glu in GM00852 had approximately 36% residual protein level (Figure 2f, lane 3) compared with wildtype enzyme in GM01653 cells (Figure 2f, lane 1). Endo-H handled samples showed Endo-Hr and Endo-Hs protein fractions at around 67 kDa and 54 kDa, respectively. The overall increase of β-Glu total enzyme by the treatments with ABX and IFG compared to the untreated condition is evident. The p.Asn370Ser β-Glu of IFG-treated cells showed a wildtype-like band pattern before and after Endo-H digestion. ABX-treated enzyme shows a characteristic, particularly pronounced Endo-Hs β-Glu band in the blot, while less Endo-Hr form was expressed.

### 2.4. ABX-Related Structures Do Not Increase Intracellular p.Asn370Ser β-Glu Activity

As mentioned above, BHX had no effect in the former chaperone study [11]. We synthesized two related BHX/ABX derivatives: 4-hydroxybromohexine (D1) contains an additional hydroxyl substituent on the cyclohexyl ring of bromohexine, yielding (1s,4s)-4-((2-amino-3,5-dibromobenzyl)(methyl)amino)cyclohexan-1-ol; in addition, the nor-derivative of bromohexine 2,4-dibromo-6-((cyclohexylamino)methyl)aniline (norbromohexine, D2) was synthesized. The latter structure lacks both the hydroxycyclohexyl and the aminomethyl group. Neither compound had a significant effect on β-Glu activity (Table 1). In silico docking revealed that the binding of ABX to β-Glu is dependent on hydrogen bonding between the 4-hydroxyl group of ABX with Asp127 and Trp381 providing the acceptor and donor sidechains, respectively [45]. We propose that additional H-bond formation between the amino group and Glu235 of the β-Glu enzyme assists in the orientation of the cyclohexane group to point inside the binding pocket (Supplementary Materials Figure S2a). The presence of a methyl group at the amine, as for BHX (and D1), prevents the interaction with Glu235, therefore switching compound orientation so that the dibromophenyl ring is pointing inside the binding pocket instead. In this case, the π-π and salt bridge interactions between the dibromophenyl ring and its positively charged NH2+ with Tyr313 and Ser345, respectively, may compensate for the lack of the hydrogen bonding interaction and, thus, explain the rather similar binding scores of the different compounds (Supplementary Materials Figure S2c). Moreover, this binding model would provide an obvious explanation for the observation that both the presence of the 4-hydroxyl group and the absence of the aminomethyl group are crucial for the intracellular chaperone effect of ABX.

### 2.5. Stabilizing Capacity of ABX and IFG on β-Glu Enzyme Defined 

**Figure 3:**
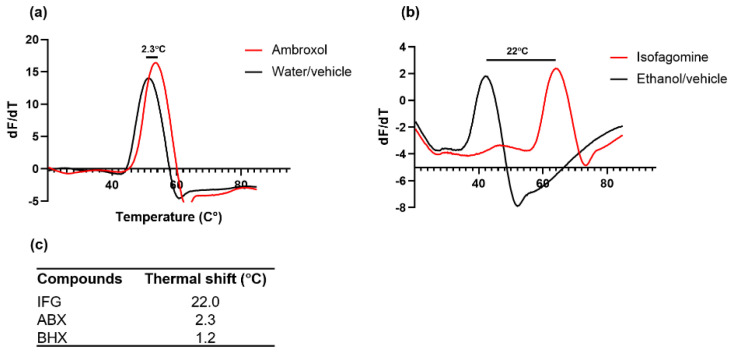
Heat-induced melting profiles of recombinant acid β-glucosidase (β-Glu) with and without ambroxol (ABX) and isofagomine (IFG). Recombinant β-Glu was mixed with different concentrations of ABX (**a**), IFG (**b**), or respective vehicle. Sypro Orange was added to a final concentration of 12.5 µM. Incrementally increased temperature over a range of 20–90 °C using the Lightcycler qRT PCR system resulted in increasing denaturation of the enzyme. (**c**) Thermal shift values were obtained by using first derivatives of raw data and were depicted for IFG, ABX, and bromohexine (BHX).

Heat-induced melting profiles of recombinant β-Glu were recorded by thermal shift at a pH value of 5.5 in the presence of 2 mM compound or respective vehicle as a control. The enzyme was heated from 20 to 90 °C in the presence of Sypro Orange, similar to the recent report for recombinant α-Gal enzyme [46]. ABX had a lesser stabilizing effect than IFG (Figure 3). This reflects the fact that ABX has lower affinity than IFG for β-Glu (K_i_ 10 µM and 0.02 µM, respectively) [1]. The denaturing temperature of ABX-treated β-Glu was 53.6 °C, which corresponded to a ΔT_M_ of merely +2.3 °C (Figure 3a,c) at a concentration in the millimolar range, whereas ABX plasma peak concentration can be considered well below 1 mM even in high-dose treatments [43]. IFG effectively stabilized the β-Glu enzyme with a ΔT_M_ of +22 °C up to 64.0 °C (Figure 3b,c). It should be noted that a pronounced solvent effect was observed with ethanol (Figure 3b). BHX showed a similar low stabilizing effect to ABX with a ΔT_M_ of +1.2 °C (Figure 3c). Hence, we show here that ABX only mildly stabilizes β-Glu in vitro, whereas the active site-specific competitive inhibitor IFG produced a significant increase in thermodynamic heat resistance of β-Glu. It is unlikely that technical aspects hamper the elucidation of stable enzyme:ABX interaction in vitro since previous reports on the inhibitory effect of ABX cover a broad range of conditions [1,7,39] with robust outcomes, indicating that the stabilizing effect cannot be enhanced dramatically by changing assay conditions. Effective PCs typically have binding affinities in the nanomolar to low micromolar range. Usually this is reflected not only in the inhibitory effect but also in a stabilization of the target enzyme in vitro while the enzyme: PC complex is formed [46,47]. Thus, the β-Glu stabilizing effect of ABX observed intracellularly is most likely not due to direct interaction between the molecules alone. Although it is conceivable that intracellular binding of the enzyme to ABX lowers the energy needed for folding so the complex can be shuttled to the lysosome, the observed incomplete processing of the enzyme in fibroblasts argues against it. Moreover, in direct binding studies, unphysiological amounts of ABX were used that exceeded obtainable ABX plasma concentration [11]. In agreement with previous results, we find that ABX is a weak in vitro stabilizer of β-Glu [24], but we cannot observe any significant gene regulatory effect of ABX on gene expression in our cells [29,45]. The formerly described beneficial effects of ABX in GD, as well as the prevention of α-synuclein protein aggregation in Parkinson’s disease [24,40], based on putative direct β-Glu stabilizing effects require reevaluation in this light. It is possible that our data may indicate an overestimation of the PC activity of ABX and thus indicate limited applicability of ABX.

In Pompe and Fabry diseases, the outcome of a combination therapy consisting of ERT and PCT utilizing the two iminosugar PCs DNJ and DGJ is expected to provide additional benefit over ERT monotherapy [48,49]. The recently published study of a combination therapy of ERT + ABX in Type III GD attended to increased β-Glu activity and encourage normalization of the lyso-Gb1 biomarker [43]. PCT can achieve effects via two different modes of action: firstly, by functionalizing and increasing the level of endogenous mutant β-Glu and secondly by stabilizing and thus increasing the half-life of the recombinant replacent enzyme of ERT. It can be assumed from our in vitro data that ABX did not act via the second mechanism because the low stabilizing effect of ABX suggests that it is not suitable as an adjuvant for ERT, whereas strong PCs such as the DGJ, DNJ, or IFG iminosugars may be an option. 

We hypothesized that a combination therapy consisting of two distinct PCTs using ABX and IFG may be an option because of the apparent partial difference in mechanism of action. A cell culture experiment showed that ABX/IFG combination-treated GM00852 and GM00372 cells displayed additive drug effects on β-Glu enzyme activity elevation compared to monotherapy with either treatment (Supplementary Materials Figure S3). Even though the exact mechanism of action of ABX remains unclear, our data suggest that additional intracellular factors are required to achieve the ABX effect, e.g., by utilizing gene regulatory function and exploiting endogenous molecular chaperones that exert a positive effect on β-Glu [19,23,24,25,26,27,50]. This peculiarity of ABX also makes it a potential drug candidate for other LSDs. Conversely, this aspect should be appreciated in future studies on the utility of ABX as PD therapy, as its putative direct effect on the correct folding of β-Glu may be overestimated.

## 3. Materials and Methods

### 3.1. IC_50_ Measurements

Imiglucerase (Cerezyme^®^, Genzyme Corporation, Cambridge, MA, USA) was a leftover from a patient who did not attend therapy in our outpatient clinic. Imiglucerase is also referred to as (recombinant) β-Glu in the text hereinafter. The powder was reconstituted in Sörensen buffer, pH 4.7, containing 0.125% sodium taurocholate on the day of the experiment. IFG, ABX, or BHX was mixed with Imiglucerase (final concentration: 0.026 µM) in a 384-well black plate (Thermo Scientific, Waltham, MA, USA) and briefly pre-incubated. An equivalent volume of 1.5 mM 4-methylumbelliferyl β-D-glucopyranoside (4-MUG, Sigma–Aldrich, Steinheim, Germany) was added and incubated for 20 min at room temperature. The reaction was stopped with glycine–NaOH buffer pH 10.5. Liberated 4-methylumbelliferone was measured (excitation 365 nm; emission 440 nm) with a Tecan Spark multimode microplate reader (Tecan, Männedorf, Switzerland). 

### 3.2. Cell Culture

Cell lines GM00372 and GM00852 (both p.Asn370Ser/84GG) and wildtype control cell line GM01653 (all obtained from Coriell Institute Cell Repository, Camden, NJ, USA) were cultured under standard conditions at 37 °C under a 5% CO_2_ atmosphere. Typically, cells were seeded in T125 flasks in DMEM and 15% FBS with the addition of penicillin/streptomycin and sub-cultured while confluence was reached. Medium was changed every 4 days. 

### 3.3. Enzyme Activity Assay 

The GM00372, GM00852, and GM01653 cells were seeded in 6-well assay plates and incubated overnight. The next day, the medium was replaced with fresh medium containing the respective compound dissolved in either dimethyl sulfoxide or water (ABX) or a mixture of ethanol and water at a ratio of 1:1 (*v*/*v*) (IFG). The fibroblasts were incubated for 5 days. After half the incubation period, the medium was replaced with fresh treatment medium. The incubation was followed by a 6 h washout period in medium without the drug. The cells were harvested by trypsination (0.25% trypsin/EDTA) and spun at 3000× *g* in a benchtop centrifuge to pellet the cells. Cell pellets were washed twice with phosphate-buffered saline and lysed by the freeze-and-thaw method in ultrapure water supplemented with 0.15% Triton X-100. The protein amount of each sample was determined by the BCA method (Thermo Scientific, Waltham, MA, USA). Lysosomal β-Glu activity was evaluated by mixing 5 µg of total protein with 3 mM of 4-MUG (Sigma–Aldrich, Steinheim, Germany) dissolved in Sorenson’s phosphate buffer (pH 5.3) containing 0.15% Triton X-100 and 0.125% taurocholate in a total volume of 30 µL in 96-well reaction plates. The reaction was incubated at 37 °C and terminated after 1 h by the addition of 0.2 mL of glycine buffer (pH 10.5). The fluorescence was measured (excitation 360 nm; emission 465 nm) in a Tecan Spark plate reader (Tecan AG, Männedorf, Switzerland). 

### 3.4. Cycloheximide Assay

Cells were plated into 6-well assay plates and treated with ABX, IFG, or the vehicle control for 5 days. Fresh treatment medium was then added with additional CHX (100 μg/mL) for the indicated period. Then, the cells were harvested in order to obtain β-Glu degradation kinetics via Western blot.

### 3.5. Western Blot

Cells were lysed on ice using RIPA buffer containing Roche complete protease inhibitor cocktail, and the protein amount of each sample was determined as above. If applicable, Endoglycosidase H (Endo-H) and peptide:N-glycosidase F (PNGase) treatment of cell lysates was carried out prior to SDS-PAGE, containing 20–30 µg of total protein, according to the company specifications (New England Biolabs, Ipswich, MA, USA). Aliquots of cell lysates were separated using Criterion precast 4–15% Tris-HCl gels (Bio-Rad, Munich, Germany) as described earlier [51]. Western blot analysis was conducted using monoclonal anti-β-Glu 2E2 antibody (Sigma–Aldrich, Steinheim, Germany) at 3 µg/mL and mouse monoclonal β-actin antibody at a 1:10,000 dilution (cat-no. A5441, Sigma–Aldrich, Steinheim, Germany) each diluted in Tris-buffered saline supplemented with 0.1% Tween-20 and 3% skim milk powder. For fluorescence detection, appropriate secondary antibodies were used (Thermo Fisher Scientific, Dreieich, Germany) for digital visualization via Odyssey Infrared Imaging system (Li-Cor Biosciences, Lincoln, NE, USA). β-Glu protein level was quantified and normalized to the internal loading control, β-actin, using the Odyssey software version 2.1. 

### 3.6. Thermal Shift Assay

Imiglucerase (final concentration: 1 mg/mL) was mixed with 10X Sypro orange dye (final concentration: 12.5 µM) in MES buffer, pH 5.5 (50 mM MES, 50 mM NaCl, and 2 mM DTE) in LightCycler^®^ 96-well white plates (Roche Applied Science, Penzberg, Germany). The compounds were added to a final concentration of 2 mM and thoroughly mixed. The plate was measured using Lightcycler 480 instrument II (Roche Applied Science, Penzberg, Germany) at an excitation wavelength of 533 nm and emission wavelength of 610 nm. The program melt curve was used to detect protein melting curves with a temperature range between 20–95 °C at 1 °C/min. T_m_ value was determined from the peak of the first derivatives of the melting curve using GraphPad Prism 5.0 (GraphPad Software, La Jolla, CA, USA).

### 3.7. Chemical Synthesis of D1 and D2

Synthesis of (1s,4s)-4-((2-amino-3,5-dibromobenzyl)(methyl)amino)cyclohexan-1-ol (D1)

#### 3.7.1. Synthesis of *tert*-Butyl-hydroxycarbamate

Following the literature procedure [52], a suspension of NH_2_OH·HCl (4.8 g, 68.7 mmol, 1.5 eq) and K_2_CO_3_ (9.5 g, 68.7 mmol, 1.5 eq) in Et_2_O (100 mL) and H_2_O (12 mL) was stirred for 1 h at ambient temperature (evolution of CO_2_). Subsequently, a solution of di-*tert*-butyldicarbonate (10.0 g, 45.8 mmol, 1.0 eq) in Et_2_O (100 mL) was added dropwise to the stirring suspension at 0 °C, and the resulting suspension was stirred at ambient temperature for 2 h. H_2_O (20 mL) was added to the reaction mixture till a clear two-phase system was obtained and the layers were separated. The aqueous phase was extracted with Et_2_O (3 × 60 mL), the combined organic layers were dried over Na_2_SO_4_ and concentrated under reduced pressure. The crude product was purified by column chromatography (SiO_2_, *n*-pentane:EtOAc = 5:1) to afford *tert*-butyl-hydroxycarbamate (6.1 g, 45.8 mmol, quant.) as a colorless solid. R*_f_* = 0.23 (*n*-pentane:EtOAc = 2:1, KMnO_4_). m.p. 51 °C. ^1^H NMR (300 MHz, CDCl_3_) δ 7.15 (s, 1H), 1.46 (s, 9H). ^13^C NMR (75 MHz, CDCl_3_) δ 159.0, 82.3, 28.3. GCMS (EI, *m*/*z*, relative intensity): 133 (0.1) [M]^+^, 59 (56), 57 (100), 43 (15), 41 (45), 39 (12). HRMS (ESI-TOF, *m*/*z*): calcd. for C_5_H_11_NO_3_ [M] 133.0733; found 133.0739. IR (ATR, neat, cm^−1^): 3356(m), 3282(w), 2979(w), 2940(w), 1679(s), 1514(m), 1459(m), 1393(m), 1367(s), 1282(s), 1252(s), 1161(s), 1119(s), 1048(m), 1014(m), 861(m), 774(m), 752(m), 517(s), 479(s).

#### 3.7.2. Synthesis of *tert*-Butyl-2-oxa-3-azabicyclo[2.2.2]oct-5-ene-3-carboxylate

*tert*-Butyl-2-oxa-3-azabicyclo[2.2.2]oct-5-ene-3-carboxylate was synthesized following a modified literature procedure [53]. CuCl (146.0 mg, 1.5 mmol, 0.2 eq) and pyridine (58.9 µL, 57.6 mg, 0.7 mmol, 0.1 eq) were added to *tert*-butyl-hydroxycarbamate (982.0 mg, 7.4 mmol, 1.0 eq) and 1,3-cyclohexadiene (843.3 µL, 709.2 mg, 8.9 mmol, 1.2 eq) in THF (30 mL). The resulting mixture was stirred vigorously for 4 h at RT and open to air. The reaction was quenched with aqueous NH_3_ (25%, 30 mL), THF was removed under reduced pressure, the aqueous phase was extracted with EtOAc (3 × 40 mL), and the combined organic layers were dried over Na_2_SO_4_ and concentrated under reduced pressure. The crude product was purified by column chromatography (SiO_2_, *n*-pentane:EtOAc = 5:1) to afford the title compound (1.4 g, 6.7 mmol, 90%) as a colorless solid. R*_f_* = 0.60 (*n*-pentane:EtOAc = 2:1, KMnO_4_). m.p. 34.5–36.5 °C. ^1^H NMR (300 MHz, CDCl_3_) δ 6.58-6.43 (m, 2H), 4.75-4.63 (m, 2H), 2.21-2.01 (m, 2H), 1.43 (s, 9H), 1.53-1.25 (m, 2H). ^13^C NMR (75 MHz, CDCl_3_) δ 157.8, 131.8, 131.6, 81.6, 70.7, 50.2, 28.3, 23.7, 20.6. GCMS (EI, *m*/*z*, relative intensity): 211 (2) [M]^+^, 111 (13), 80 (39), 79 (66), 78 (12), 77 (12), 57 (100), 41 (30), 39 (12). HRMS (ESI-TOF, *m*/*z*): calcd. for C_11_H_17_NO_3_ [M] 211.1203; found 211.1208. IR (ATR, neat, cm^−1^): 2979(m), 2937(w), 1673(s), 1615(w), 1455(m), 1395(s), 1360(s), 1293(m), 1253(s), 1162(s), 1125(m), 1110(s), 1076(s), 1049(s), 993(m), 957(m), 918(m), 879(s), 849(m), 818(m), 797(m), 745(s), 713(s), 680(m), 666(m), 614(m), 492(m), 429(m).

#### 3.7.3. Synthesis of 3-*N*-Methyl-2-oxa-3-azabicyclo[2.2.2]oct-5-ene

3-*N*-methyl-2-oxa-3-azabicyclo[2.2.2]oct-5-ene was synthesized following a modified literature procedure [54]. A solution of *tert*-butyl-2-oxa-3-azabicyclo[2.2.2]oct-5-ene-3-carboxylate (6.3 g, 30.0 mmol, 1.0 eq) in tetrahydrofuran (50 mL) was added dropwise to a stirring suspension of LiAlH_4_ (2.3 g, 60.0 mmol, 2.0 eq) in tetrahydrofuran (50 mL) at 0 °C. The ice-bath was removed, and the reaction mixture was stirred at ambient temperature for 1 h. Afterwards, the reaction mixture was cooled to 0 °C and quenched carefully with methanol and aqueous K_2_CO_3_ (pH = 10). The insoluble material was filtered off, and volatiles were removed under reduced pressure. The crude product was dissolved in dichloromethane and the aqueous phase was extracted with dichloromethane (5 × 50 mL). The combined organic layers were dried over Na_2_SO_4_, and the crude product was purified by column chromatography (SiO_2_, CH_2_Cl_2_:EtOAc = 20:1 + 2% NEt_3_→CH_2_Cl_2_:EtOAc = 5:1 + 2% NEt_3_), affording 3-*N*-methyl-2-oxa-3-azabicyclo[2.2.2]oct-5-ene (2.6 g, 20.7 mmol, 69%) as a brown oil. R*_f_* = 0.60 (CH_2_Cl_2_:MeOH = 10:1, KMnO_4_). ^1^H NMR (300 MHz, CDCl_3_) δ 6.66-6.56 (m, 1H), 6.33-6.22 (m, 1H), 4.39-4.28 (m, 1H), 3.57-3.46 (m, 1H), 2.37 (s, 3H), 2.11-1.93 (m, 2H), 1.48-1.18 (m, 2H). ^13^C NMR (75 MHz, CDCl_3_) δ 132.8, 129.4, 68.0, 56.0, 44.8, 23.2, 22.4. GCMS (EI, *m*/*z*, relative intensity): 125 (10) [M]^+^, 124 (92), 111 (10), 97 (17), 96 (17), 95 (20), 83 (10), 81 (13), 80 (91), 79 (100), 78 (24), 77 (49), 69 (12), 68 (16), 67 (37), 57 (13), 55 (15), 54 (13), 46 (70), 43 (11), 41 (23), 39 (17). HRMS (ESI-TOF, *m*/*z*): calcd. for C_7_H_11_NO [M] 125.0841; found 125.0849. IR (ATR, neat, cm^−1^): 3051(w), 2953(m), 2877(m), 1452(m), 1431(m), 1368(m), 1310(w), 1220(w), 1179(m), 1164(m), 1087(w), 1058(m), 998(m), 986(m), 955(s), 931(s), 853(m), 816(m), 801(m), 718(s), 704(s), 654(s), 569(m), 478(m), 438(s).

#### 3.7.4. Synthesis of (*Syn*)-4-(methylamino)cyclohexan-1-ol

Following a modified literature procedure [55], a solution of (1*R*,4*S*)-3-methyl-2-oxa-3-azabicyclo[2.2.2]oct-5-ene 22 (1.0 g, 8.0 mmol, 1.0 eq) in dry methanol (40 mL) was hydrogenated in the presence of 5% Pd/C (1.7 g, 0.8 mmol, 0.1 eq) at RT under atmospheric pressure of H_2_ (balloon) for 24 h. The catalyst was removed by filtration through celite, and the crude product was purified by column chromatography (SiO_2_, EtOAc:MeOH = 5:1 + 2% NEt_3_) and sublimation (0.02 mbar, 50 °C) to afford (*syn*)-4-(methylamino)cyclohexan-1-ol (227.3 mg, 1.8 mmol, 22%) as a colorless solid. R*_f_* = 0.06 (EtOAc:MeOH = 5:1 + 2%NEt_3_, KMnO_4_). m.p. 84–85 °C. ^1^H NMR (300 MHz, CD_2_Cl_2_) δ 3.87-3.73 (m, 1H), 2.59 (s, 1H), 2.49-2.35 (m, 1H), 2.37 (s, 3H), 1.78-1.43 (m, 8H). ^13^C NMR (75 MHz, CD_2_Cl_2_) δ 67.3, 57.1, 33.7, 31.6, 27.5. GCMS (EI, *m*/*z*, relative Intensity): 129 (12) [M]^+^, 71 (17), 70 (100), 57 (38), 42 (7), 30 (4). HRMS (ESI-TOF, *m*/*z*): calcd. for C_7_H_15_NO [M] 129.1154; found 129.1162. IR (ATR, neat, cm^−1^): 3261(m), 3087(m), 2974(w), 2936(s), 2849(s), 2785(s), 2676(w), 1563(w), 1499(w), 1430(m), 1368(s), 1353(m), 1330(m), 1309(w), 1284(m), 1261(s), 1218(m), 1137(m), 1119(s), 1090(m), 1060(m), 1031(m), 974(s), 902(s), 824(m), 763(m), 686(m), 651(m), 507(m), 494(m), 464(w), 416(m).

#### 3.7.5. General Procedure for the Syntheses of 2,4-Dibromo-6-((cyclohexylamino)methyl) aniline-derivatives (GP1)

2-amino-3,5-dibromobenzaldehyde (1.0 eq) and cyclohexylamine-derivative (1.5 eq) were dissolved in dry iPrOH (0.1 M) in a preheated flask under Ar and at 40 °C. Then, Ti(OiPr)_4_ (2.0 eq) was added dropwise to the stirring reaction mixture. After the reaction was continued at 40 °C for 24 h, NaBH_4_ (2.5 eq) was added to the reaction solution in portions and stirred at 40 °C for an additional 24 h. After completion, H_2_O was added to the solution to quench the reaction and precipitate a large amount of solid, which was removed by suction filtration. The solvent was evaporated, the aqueous phase adjusted to pH = 10, extracted with EtOAc (3 × 20 mL), dried, and purified by column chromatography.

#### 3.7.6. Synthesis of (1s,4s)-4-((2-Amino-3,5-dibromobenzyl)(methyl)amino)cyclohexan-1-ol (D1)

Following the general procedure GP1, 2-amino-3,5-dibromobenzaldehyde (215.9 mg, 0.8 mmol, 1.0 eq) and *cis*-4-(methylamino)cyclohexan-1-ol (150.0 mg, 1.2 mmol, 1.5 eq) were first treated with Ti(OiPr)_4_ (469.5 µL, 439.9 mg, 1.6 mmol, 2.0 eq) and then with NaBH_4_ (73.2 mg, 1.9 mmol, 2.5 eq). Purification by column chromatography (SiO_2_, *n*-pentane:EtOAc = 5:1) yielded the title compound D1 (190.8 mg, 0.5 mmol, 63%) as a yellow viscous oil. R*_f_* = 0.25 (*n*-pentane:EtOAc = 1:1, KMnO_4_). ^1^H NMR (300 MHz, CDCl_3_) δ 7.46 (d, *J* = 2.2 Hz, 1H), 7.04 (d, *J* = 2.2 Hz, 1H), 5.45 (s, 2H), 3.99 (p, *J* = 3.2 Hz, 1H), 3.60 (s, 2H), 2.50-2.34 (m, 1H), 2.14 (s, 3H), 1.93-1.69 (m, 4H), 1.66-1.41 (m, 4H). ^13^C NMR (75 MHz, CDCl_3_) δ 144.1, 133.2, 131.8, 110.2, 108.2, 65.8, 61.2, 58.0, 36.6, 32.2, 22.1. GCMS (EI, *m*/*z*, relative Intensity): 394 (7) [M]^+^, 392 (13) [M]^+^, 390 (7) [M]^+^, 335 (10), 333 (20), 331 (10), 307 (10), 305 (21), 303 (11), 295 (32), 291 (34), 266 (28), 335 (10), 264 (57), 262 (30), 142 (15), 128 (29), 104 (16), 77 (10), 70 (100), 57 (10), 44 (14), 42 (15). HRMS (ESI-TOF, *m*/*z*): calcd. for C_14_H_20_ON_2_^79^Br_2_ [M] 389.9937; found 389.9928; calcd. for C_14_H_20_ON_2_^79^Br^81^Br [M] 391.9916; found 391.9925. IR (ATR, neat, cm^−1^): 3429(w), 3258(w), 2926(m), 2850(m), 2792(w), 1604(m), 1575(w), 1550(m), 1459(s), 1379(m), 1356(m), 1291(m), 1256(m), 1232(m), 1196(m), 1123(m), 1100(m), 1072(m), 1031(s), 987(m), 958(s), 929(m), 905(m), 885(m), 856(s), 816(m), 736(m), 679(s), 646(s), 551(s), 444(s).

#### 3.7.7. Synthesis of 2,4-Dibromo-6-((cyclohexylamino)methyl)aniline (D2)

Following the general procedure GP1, 2-amino-3,5-dibromobenzaldehyde 18 (253.1 mg, 0.9 mmol, 1.0 eq) and cyclohexylamine (156.9 µL, 135.0 mg, 1.4 mmol, 1.5 eq) were first treated with Ti(OiPr)_4_ (550.5 µL, 515.8 mg, 1.8 mmol, 2.0 eq) and then with NaBH_4_ (85.8 mg, 2.3 mmol, 2.5 eq). Purification by column chromatography (SiO_2_, *n*-pentane:EtOAc = 10:1) yielded the title compound D2 (292.7 mg, 0.8 mmol, 89%) as a light yellow oil. R*_f_* = 0.13 (*n*-pentane:EtOAc = 10:1, UV). ^1^H NMR (300 MHz, CDCl_3_) δ 7.46 (d, *J* = 2.3 Hz, 1H), 7.09 (dt, *J* = 2.3, 0.7 Hz, 1H), 5.41 (s, 2H), 3.79 (t, *J* = 0.5 Hz, 2H), 2.54-2.34 (m, 1H), 1.98-1.83 (m, 2H), 1.79-1.66 (m, 2H), 1.65-1.55 (m, 1H), 1.39-0.97 (m, 5H). ^13^C NMR (75 MHz, CDCl_3_) δ 144.1, 133.2, 131.4, 127.1, 110.5, 108.3, 56.2, 50.3, 33.6, 26.2, 25.0. GCMS (EI, *m*/*z*, relative Intensity): 364 (8) [M]^+^, 362 (15) [M]^+^, 360 (8) [M]^+^, 293 (13), 291 (27), 289 (15), 281 (49), 280 (10), 279 (100), 277 (52), 266 (42), 264 (87), 262 (45), 183 (10), 112 (12), 104 (16), 293 (13), 98 (67), 56 (18), 55 (12), 41 (11). HRMS (ESI-TOF, *m*/*z*): calcd. for C_13_H_18_N_2_^79^Br^81^Br [M] 361.9811; found 361.9806; calcd. for C_13_H_18_N_2_^81^Br_2_ [M] 363.9790; found 375.9799. IR (ATR, neat, cm^−1^): 3435(w), 3265(w), 2922(s), 2849(m), 1606(m), 1577(w), 1552(m), 1459(s), 1408(m), 1371(m), 1346(m), 1284(w), 1242(m), 1195(m), 1144(w), 1111(m), 1077(s), 988(w), 956(m), 889(m), 858(s), 818(m), 799(m), 758(m), 682(s), 621(s), 551(s), 509(m), 448(s).

### 3.8. Statistical Evaluation

All statistical analyses were calculated using GraphPad Prism, versions 5.0 and 9.1.0 (for Windows).

## 4. Conclusions

Pharmacological chaperones merit attention as candidate drugs for the treatment of Gaucher disease, specifically because they promise hope for the treatment of Type III GD. The present report juxtaposes the specific properties of the effects of ambroxol with those of the known PC isofagomine using molecular and biochemical examinations. ABX increases β-Glu activity measured in lysates of GD patient fibroblasts, quantitatively indistinguishable from IFG. Nevertheless, the ability of ABX to stabilize recombinant β-Glu in vitro is limited compared with IFG, and cellular stabilization of mutant β-Glu is also less effective, as verified by evidence of incomplete cellular processing.

Closely related structural ABX analogues show no chaperone effect on β-Glu, and in silico docking reveals a possible position reversal in the binding pocket of compounds with an aminomethyl group, suggesting that ABX and derivatives might enter the active site in reverse position, with similar binding enthalpies but different stabilizing efficiency. Thus, the efficacy of ABX as a GCase binder and stabilizer in GD (and likely as a protein aggregation inhibitor for Parkinson’s disease) must be questioned. Our data emphasize the possibility that ABX function may be due to an as-yet-unexplained mode of action in the cellular context.

## Figures and Tables

**Table 1:** Maximum acid β-glucosidase (β-Glu) activity increases in cells.

Cmpd	β-Glu Activity [FC] (GM00852)	β-Glu Activity [FC] (GM00372)	β-Glu Activity [FC] (GM01653)
ABX	2.0 ± 0.15 ^†^ (40 µM), ****	1.6 ± 0.12 (40 µM), **	1.3 ± 0.12 (20 µM), n.s.
IFG	2.4 ± 0.17 (40 µM), **	1.6 ± 0.16 (100 µM), **	1.3 ± 0.17 (100 µM), n.s.
BHX	1.1 ± 0.09 (40 µM), n.s.	n/a	n/a
D1	1.3 ± 0.16 (40 µM), n.s.	n/a	n/a
D2	1.2 ± 0.05 (40 µM), n.s.	n/a	n/a

Experiments were repeated (*n* = 6; except ^†^ *n* = 12). Statistical analysis was carried out as specified in the legend for Figure 2a–c. Asterisks indicate significance level compared to the untreated state: **, *p* < 0.01; ****, *p* < 0.0001; n.s., not significant; n/a, not analyzed.

## Data Availability

Data is contained within the article and supplementary material.

## Supplementary Materials

The following supporting information can be downloaded at: https://www.mdpi.com/article/10.3390/ijms23073536/s1.
