# Supplementary figures and images for: Mechanistic Insight into the Mode of Action of Acid β-Glucosidase Enhancer Ambroxol
Source: Int J Mol Sci. 2022 Mar 24;23(7):3536. doi: 10.3390/ijms23073536 (PMC8998264; doi:10.3390/ijms23073536)

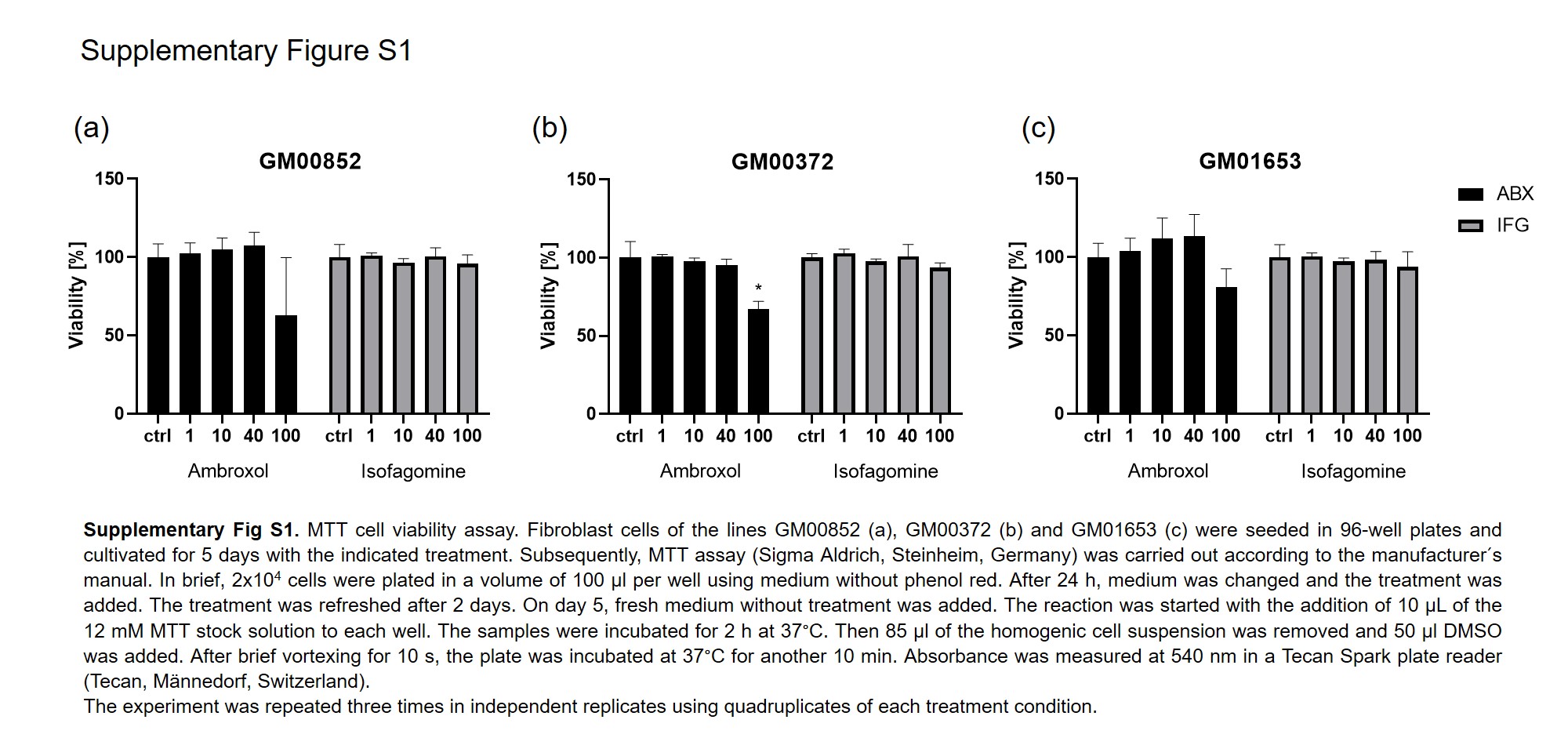

Supplement: Supplementary file 1 [file ijms-23-03536-s001.zip › Supplementary File S1.jpg]

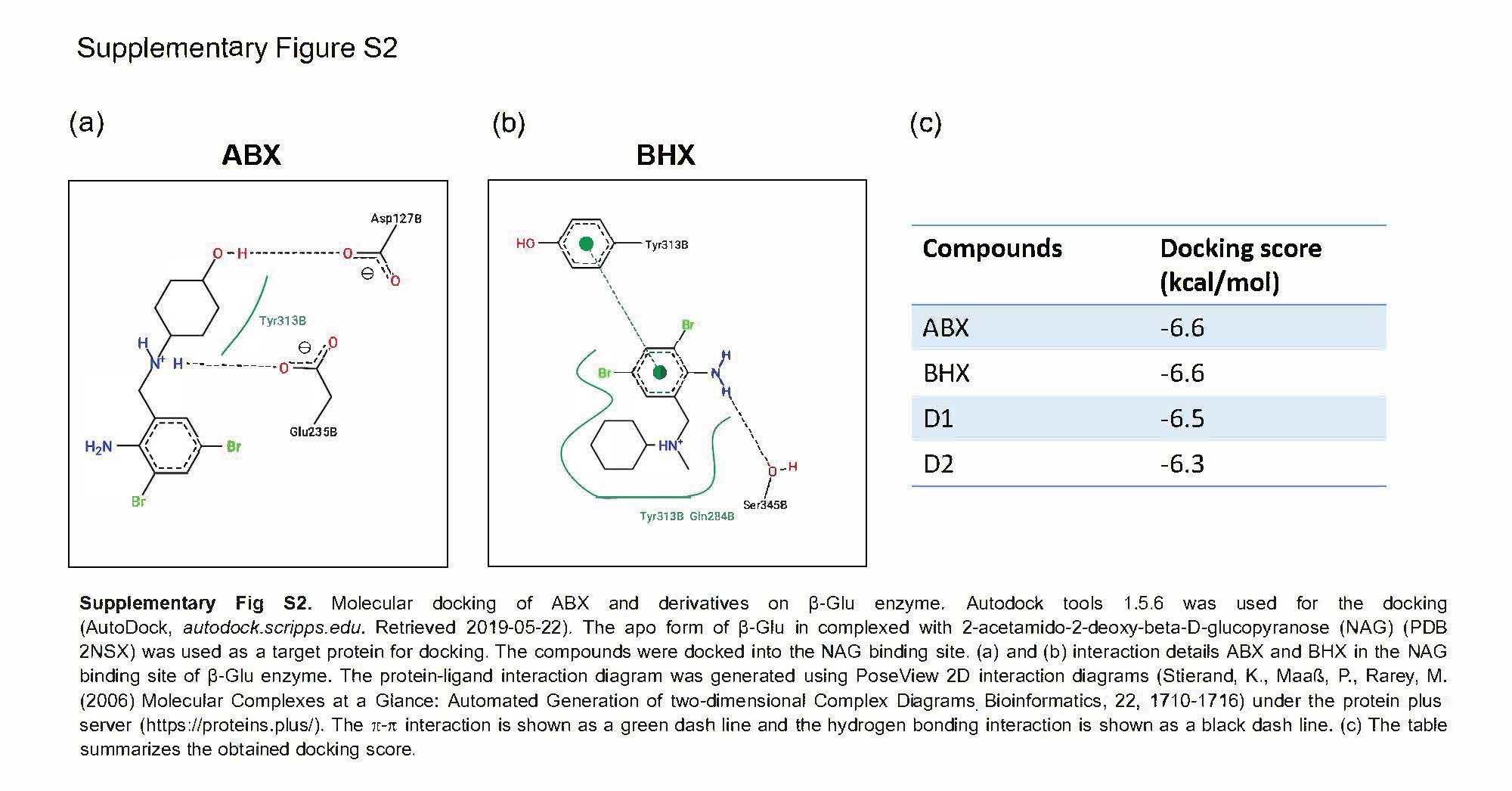

Supplement: Supplementary file 1 [file ijms-23-03536-s001.zip › Supplementary File S2.jpg]

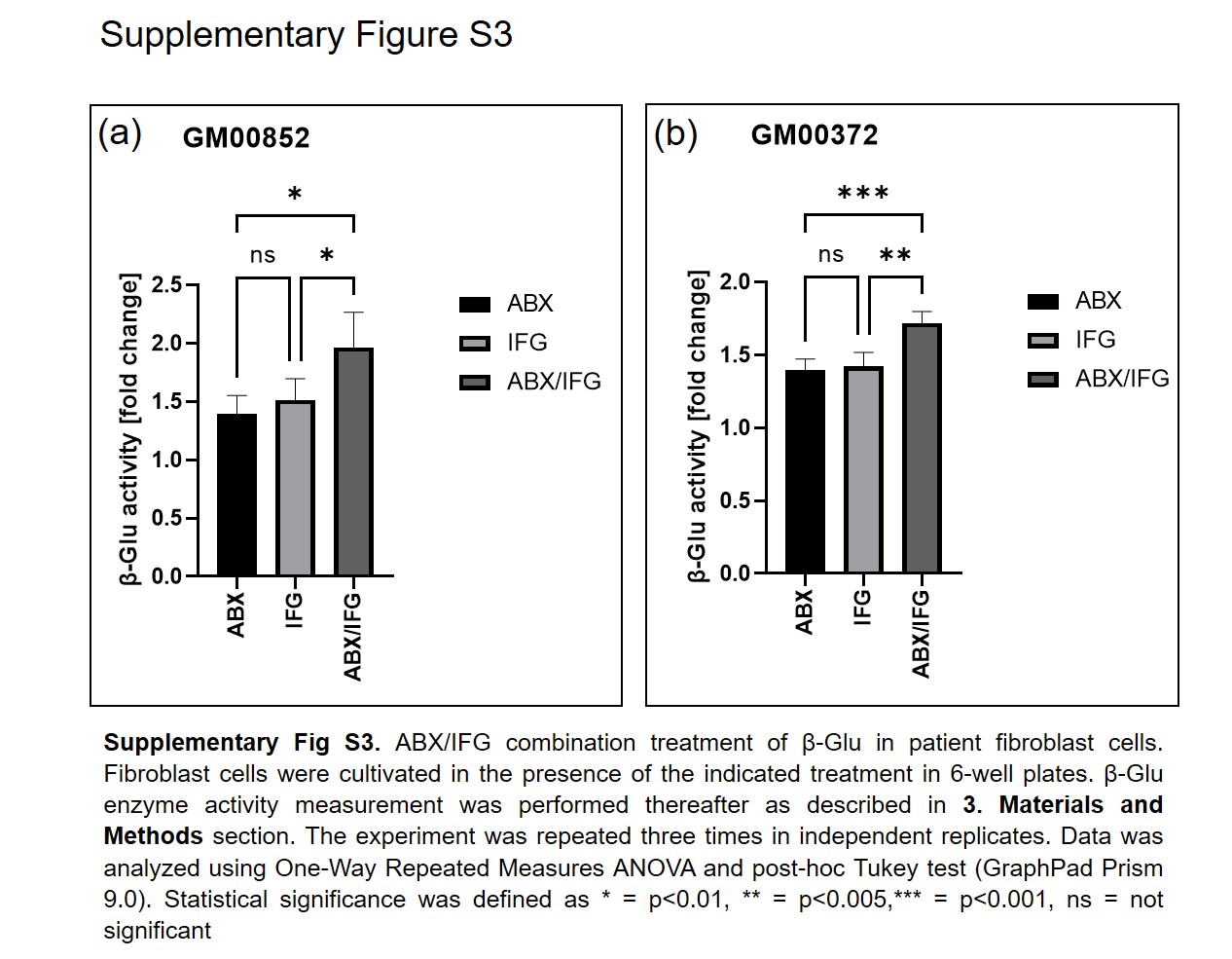

Supplement: Supplementary file 1 [file ijms-23-03536-s001.zip › Supplementary File S3.jpg]
